# When you wish upon a (GWP) star: Environmental governance and the reflexive performativity of global warming metrics

**DOI:** 10.1177/03063127221134275

**Published:** 2022-11-15

**Authors:** George Cusworth, Jeremy Brice, Jamie Lorimer, Tara Garnett

**Affiliations:** 1 University of Oxford, Oxford, UK; 2 Hertford College, Oxford, UK

**Keywords:** metrics, performativity, climate change, environmental governance, reflexive modernization

## Abstract

The metrics used in environmental management are performative. That is, the tools deployed to classify and measure the natural world interact with the things they were designed to observe. The idea of performativity also captures the way these interactions shape or distort the governance activities that metrics are used to inform. The performativity of metrics reveals how mundane practices of measurement and auditing are inscribed with substantial power. This has proven particularly true for the global warming metrics, like GWP100, that are central to the management of anthropogenic climate change. Greenhouse gases are materially heterogenous, and the metrics used to commensurate their various warming impacts influence the distribution of both culpability and capital in climate policy and markets. The publication of a new warming metric, GWP* (or GWP Star), has generated a modest scientific controversy, as a diverse cast of stakeholders recognize this performativity seek to influence the metrological regime under which they live. We analyse this controversy, particularly as it unfolded in the fractious discourse around sustainable food and farming, to develop the concept of *reflexive performativity*: where actors are anticipatory and strategic in their engagement with the metrics that are used to govern their lives. We situate this idea in relation to, and in tentative evidential support of, the concept of reflexive modernization.

In 1577, the peasants in the Polish villages of Iskrzynia and Krościenko complained to King Stephan Báthory about the bushels their lords used to gather tithes. The peasants claimed that the local lords increased the size of the containers employed to measure out their lordly due, thus increasing the portion of the harvest diverted to the manor. The King issued a decree forcing the lords to revert to their old measures and to refrain from using such underhanded tricks in the future (Kula, 1986). For centuries, this vignette tells us, the material objects – containers, weight measures, and rods – that were supposed to provide an even-handed epistemic framework for commerce and have been subject to the same power structures they were designed to counter.

What has changed in the past 500 years? Certainly, disagreements rarely concern crude issues like the magnitude of a tonne of grain or a hectare of cropland. Nevertheless, arguments about metrics persist, arising because tools of measurement shape the worlds they were, in theory, designed only to record. These observations will be familiar to readers of this journal (e.g. MacKenzie, 2003), particularly as they relate to questions of environmental governance (Mathews, 2014). Lists of threatened species, for instance, created to provide a policy-relevant but apolitical database about the extinction risk faced by different species, overdetermine the distribution of funding in conservation work (Bowker, 2000a; Braverman, 2017). Management interventions based on satellite imagery privilege institutional and commercial rather than local preferences for which types of land cover ought to receive protection (Robbins, 2001). Environmental subsidies indexed to a particular metric like carbon sequestration can have unintended impacts on the communities proximate to the intervention (Carton, 2020) or on other environmental objectives like biodiversity conservation (Phelps et al., 2012).

The idea of performativity runs through these analyses, capturing the fact that acts of measurement impact the thing being measured, or even mould the world into a truer reflection of the theory designed to describe it (MacKenzie, 2003). In this performative understanding, the real may start to ‘converge’ (Bowker, 1994) with its representation (Winner, 1977). In the context of climate change governance, the metrics used to commensurate the warming impacts of different climate forcing agents (like methane and carbon dioxide) can have substantive sway on the perceived warming culpability of different polluters (Cooper, 2015) and the finances that flow through emission trading schemes (MacKenzie, 2009).

This article uses an index of ‘warming potential’ to illustrate a phenomenon that is under-theorized in the literature: how the performativity of a metric becomes subject to engagement by those it will come to affect. We are interested in how non-technical and non-scientific actors to weigh in on seemingly arcane discussions about metric design, knowing that such decisions will shape the worlds they have to inhabit. This phenomenon can be seen in Kula’s lively feudal parable above. The containers used to measure out tithes had such direct influence over the lives of the peasants that they sought legal recourse to ensure that the metrological regime under which they had to live was fair. Our project has its roots in the studies of organizational management (Pollock et al., 2018; Power, 1994; Strathern, 2000) that have documented the way interested parties respond to systems of workplace and economic auditing. We term this phenomenon, where actors and institutions reflexively grapple with the performativity of metrics and look to shape the metrological regime in which they dwell, *reflexive performativity*.

Our first aim is to provide the conceptual terminology and dedicated case study necessary to depict this phenomenon, which has, to date, been only briefly noted as part of a broader package of the ways actors engage with infrastructures of measurement and evaluation (e.g. Pollock et al., 2018; Sauder & Fine, 2008). To achieve this aim, we illuminate when and why the process of reflexive performativity takes hold. We describe the phenomenon of reflexive performativity and show what the concept can do for scholars interested in the link between measurement and governance, through a close reading of the controversy that has arisen from the publication of a new Global Warming Potential (GWP) metric, GWP* (sometimes written GWP Star). We attend to the political work the metric is being made to undertake and scrutinize the wishes being made in the name of this new (GWP) star. Our study provides new vistas on the research agendas that Pollock et al. (2018) and Power (2015) outline regarding the ways actors respond to and resist audit systems.

The secondary ambition of the paper is to contribute to the literature on how the issue of climate change is co-produced as a matter of political and technical concern by scientific organizations (Lahn, 2021; Mahony & Hulme, 2018) and the way the metrics used to organize climate governance exert their influence on mitigation activities (Cooper, 2015; Lovell et al., 2013; MacKenzie, 2009; Ormond & Goodman, 2015), particularly in the agricultural sector (Cooper & Rosin, 2014). Warming metrics are designed to compare the relative warming impact of different greenhouse gases (GHGs), commensurating their differing properties and behaviours in the atmosphere into a common *carbon dioxide equivalence* unit. They are needed to provide a system of measurement to organize emissions budgets and cap-and-trade schemes (MacKenzie, 2009). The way the standard GWP100 metric goes about this, the authors of GWP* argue, produces misleading evaluations of the climate forcing impacts of different gases and polluters (Allen et al., 2018).

To unpack the GWP* controversy and to understand what general lessons can be extracted from this case, we apply Cooper’s (2015) *critical metrological approach.* Cooper encourages social scientists to pay close attention to the specifics of the metric in question (here, the differing mathematics GWP100 and GWP* employ to commensurate warming impacts) and to take seriously the ‘social, political, and scientific conditions under which measurement and commensuration occur, as well as the consequences or effects of these processes’ (Cooper, 2015, p. 1788). To answer this call, we focus our attention on the arena where the ‘politics and consequences’ of this new metric are most pronounced: sustainable food and livestock agriculture. As a result of its mixed emissions profile, animal agriculture is particularly exposed to the ways in which different GHGs are made commensurate. Many of the actors participating in the debate around GWP* have done so to articulate different versions of food system sustainability. One of the most prominent claims emerging from the controversy is that the warming impacts of livestock systems have been over-estimated by calculations predicated on the standard GWP100 metric, and that now-familiar injunctions to reduce red meat consumption for environmental reasons need to be challenged. This story of metrics, animal agriculture, and climate change offers an account of the performativity of metrics, but also to explore the phenomenon of reflexive performativity, too.

In the following section we outline the paper’s theoretical framework, introducing the study of metrics in the context of environmental and climate governance, the concept of performativity and Cooper’s critical metrological approach. We then describe the methods used to undertake our analysis. This is followed by a technical description of GWP* and the modest controversy its publication has precipitated. In the main analysis, we present the phenomenon of reflexive performativity and situate it in relation to the influential account of *reflexive modernization* offered by Beck, Giddens and Lash (Beck et al., 1994).

## Studying metrics

Metrology is concerned with the practice of measurement. It is interested in methods of classification and of standardization into taxonomies, and in the instruments (calculative, theoretical, or physical) involved in acts of measurement and commensuration (Cooper, 2015). Metrics – the general term used to refer to the tools involved in recording and standardization – have been central to the outward expansion of the epistemological authority of science (Latour, 1983). They provide the standards that enable knowledge produced in the confines of a laboratory to travel and to acquire credibility in new contexts.

A substantial body of social science research has scrutinized the design and application of metrics in environmental governance. Much of this research is animated by a suspicion about the roles that metrics have played in the expansion of market logics into the worlds of climate and conservation management. Market-based tools of environmental governance like subsidy schemes, emission credits, and payments for ecosystem services all rely on metrological regimes that allow complex ecosystems to be made into stable, enumerated, and codified objects suitable for commodification (e.g. Castree, 2003; McElwee, 2017; Prudham, 2009; Robertson, 2012; Turnhout et al., 2014). Forests, in this framework, are reduced to a measurement of above-ground biomass (Edstedt & Carton, 2018), while the health of a river delta becomes known through populations of select indicator species (Lakoff, 2016). In this model, metrics homogenize and flatten (Robertson, 2000) things that were previously heterogenous and ‘thick’ with layered meanings (Alder, 1998).

Within this literature, there is a particular body of work interested in the epistemological contortions needed to bring GHG emissions into the politics of climate governance. Climate change is being caused by a range of GHGs, each of which exerts a different level of radiative force (the amount of solar energy the gases trap in the atmosphere) over a different period of time. Warming metrics, with GWP100 being the standard metric in climate governance (Lynch, Cain et al., 2020), commensurate this heterogeneity to express their warming impact in a shared *carbon equivalence* unit, CO_2_e. The metric thus allows an emission or offset of one gas originating in one part of the world to be made fungible (and tradeable) with an emission or offset of a different gas coming from some distant location (MacKenzie, 2009). The desire to commensurate GHGs in this way is, in part, bound up with a preference for conceiving of climate change as an economic problem (of negative externalities to be internalized) to legitimate and enable capital-friendly solutions like offset markets and emissions trading schemes (Cooper, 2015; Lohmann, 2010; Lovell et al., 2013).

The idea of performativity is central to understanding the controversy that has arisen from the publication of the GWP* metric. The term is used to capture similar but not identical phenomena. In philosophy and linguistics, performativity originally referred to the ways certain speech acts have more-than-semantic implications. For example, the words ‘I do’ uttered by the bride and groom at a wedding ceremony, or ‘out’ by a cricket umpire do something more than just relay meaning (Austin, 1962): They confirm the marriage in the eyes of the law and force the batsman from the crease. Butler’s (1990) use the performativity of language and behaviour makes it more diffuse, its effects accumulating over time and space rather than happening with a specific utterance. Gender categories, for example, are created by the performative powers of gender-script behavioural and linguistic tropes.

STS scholars have applied performativity to the production of scientific knowledge, pointing out that ‘analytical systems that link different concepts’ may not merely ‘explain or predict empirical phenomena’ (Marti & Gond, 2018, p. 489), but also bring some new reality into existence. In Garud and Gehman’s (2019, p. 2) formulation, performativity ‘emphasizes the constitution of new worlds through their articulation’. The idea troubles a simplistic conceptualization of the activities of science and measurement as purely representational and engaged in the business of describing independent facts (Pickering, 1994).

Of particular interest for this paper is the performativity of metrics. Metrics now shape spheres of life as diverse as the economy, employment, personal fitness regimes, and the distribution of justice (Beer, 2016; Espeland & Stevens, 2008; Saetnan et al., 2011; Shore & Wright, 2015). Although the performative power that metrics have is not universal (Pollock et al., 2018), metrics rarely simply record the world from a distance; they interact with it. In some cases, actors and institutions falling under the jurisdiction of a metric shift their behaviours (consciously or not) to satisfy, appease, or game the metrics (Espeland & Stevens, 2008; Sauder & Espeland, 2009). In other cases, metrics structure the values used to assess the thing being measured, leading Alldred and Miller (2007) to wonder whether we measure what we value or value what we measure. Landmark studies, like Stiglitz et al.’s (2009) cultural and economic analysis of the GDP measure, have highlighted the perverse outcomes that are produced through attempts to index complex socio-cultural phenomena (like wellbeing) to widely used metrics (like a nation’s wealth as a function of its population). We use Beer’s (2016) term ‘metric power’ throughout this paper to refer to the influence metrics have on the objects they purport to observe.

As the research around the idea of governmentality (Dean, 2009; Murray Li, 2007; Rose et al., 2006) has shown, the mundane tools of measurement and calculation are the covert means through which powerful actors arrange the social and economic order. Scholars developing the concepts of measurementality (Turnhout et al., 2014) and environmentality (Agrawal, 2005) have used the same Foucauldian framings to study the power inscribed in the tools of measurement used in environmental governance. In Lidskog’s (2014, p. 671) words ‘how an environmental problem is represented and measured – provided this representation is seen as scientifically sound and the policy community deems it valid – has great implications for its regulation’.

The idea of performativity has had particular traction for those studying governance of anthropogenic warming. As its impacts are largely diffuse, distant, or yet to come, the issue of climate change is infused with an aura of uncertainty (Anderson, 2010), risk (Bulkeley, 2001), and even apocalypse (Swyngedouw, 2010). As a result, a performative choreography of measurements describing emission rates and temperature change is needed to make the impacts of climate change sufficiently real to generate interest, to ‘create’ offset credits that can be brought to market, or otherwise make it an issue of concern (Lansing, 2012; Lohmann, 2014). Vulnerability to climate change (and thus the receipt of funds for its mitigation) can, for example, be performatively conjured up by a cast of community stakeholders, politicians, and adaptation or finance technocrats (Webber, 2013). Schemes to incentivize sequestration through reforestation performatively create their emission offset credits by calculations of measurement, reporting and verification (Gupta et al., 2012). Efforts to encourage more ambitious mitigation have followed suit, with activists organizing their actions around estimations of the warming implications of business-as-usual scenarios, using emissions calculations to performatively bring about social change (Beuret, 2017).

The performativity of systems of measurement and auditing have also been studied in food systems. Metrics and sustainability evaluations have become interwoven into the promissory narratives that orbit alternative food systems (Maye & Duncan, 2017) and novel foodstuffs like cultured meat (Stephens et al., 2018). Recognition of the power of food system sustainability quantification was manifest in the heated discussion around the proposal for a ‘IPCC for food’ at the 2021 UN Food Systems Summit. Many stakeholders were concerned that the body, which was proposed to provide technical and scientific input on food governance, would be used to promote a science-policy agenda that will exclude smaller stakeholder groups and prioritize high-tech and business-orientated food system solutions (Clapp et al., 2021). The controversy it sparked can be read as mass participation in negotiations around the most appropriate, powerful, and fair way to arrange metric power in the food system.

The most relevant work to this study comes from the researchers exploring the performativity of warming metrics in climate governance. GHGs are materially heterogenous (Liu, 2017) and choosing a commensuration always involves the exclusion of plausible alternative methodologies (Cooper, 2015). Different metric designs make the warming impacts of GHGs produce varying evaluations of the relative severity of the same polluting behaviours. As these metrics mediate political and cultural engagement with the warming responsibility of different actors, they go on to shape the interventions designed to incentivize warming mitigation (MacKenzie, 2009) and the commercial atmosphere in which retailers vie for consumers (Ormond & Goodman, 2015). Warming metrics like GWP100 are thus performative: They shape the mitigating activities that underpin sustainability labelling (Ormond & Goodman, 2015), offset markets (MacKenzie, 2009), carbon accounting (Ascui & Lovell, 2011), activism (Beuret, 2017), and green finance (Lohmann, 2014). As with the literature describing the performativity of the metrics used in other environmental spheres (e.g. Braverman, 2017; Robbins, 2001), the implication is that warming metrics’ ability to provide an apolitical epistemological framework through which the activities of climate governance can be conducted is compromised (Cooper, 2015). To use Callon’s (1998) terminology, their ability to ‘cool’ (provide an epistemological frame that can act as a consensus point for multiple stakeholders) a ‘hot’ situation (in which everything, including the scientific knowledge about the situation, becomes controversial) like climate governance loses purchase.

It is against the backdrop of this scholarship that Cooper (2015) presents his framework for critical metrology. He argues that markets, including those for emissions, offsets, and ecosystem services, always favour some industries and individuals over others. And, as these markets rely on a metrological regime to render complex natural systems (like the climate forcing impacts of GHGs in the atmosphere) into fungible units (like CO_2_e), metrics are always political. There is nothing inevitable about what a metric choose to count, or how it chooses to standardize different things (like the warming impact of different GHGs). And there is nothing straightforward about the way metrics structure the activities designed to manage the environmental issue at hand.

While there is a substantial body on the epistemological work that metrics do to facilitate the activities of environmental and climate management, for Cooper (2015), not enough of this work has grappled with the particulars of metrological procedures, how are selected, and how their selection influences the governance of the thing in question (see also Mahony & Hulme, 2018). He calls for the social scientist to pay close attention to all these specifics. It is in this critical metrological spirit that we look to make our own research contribution: paying close attention to the design of GWP* relative to GWP100, the conditions under which it has been presented, and the active political life it is now living.

## Methods

This analysis is based on interviews with 28 individuals from organizations central to the flow of ideas around food and farming, including advocacy groups, industry bodies, scientists, civil servants, and NGOs. All interviewees were UK-based and spoke primarily in relation to UK food and farming. In the purposeful selection of our interviewees, we were particularly interested in those who speak concerning livestock farming and for meat and dairy consumption. Not least because of already-heightened tensions in the sector, GWP* has seen particular uptake among those engaged in the emotive disagreements relating to the places meat and dairy ought to have in sustainable and healthy diets. In addition to these interviews, we attended tens of hours of industry conferences, webinars and seminars and other events on the topics of GWP*, agricultural emissions, and food sustainability. A more general immersion in the controversy around this metric was achieved by reading published communications from NGOs, sector bodies, and industry journalism on those same topics. The data collection took place from winter 2020 to summer 2021.

## GWP*, GWP100 and the calculation of agricultural emissions

Under GWP100, different GHGs are standardized by comparing the warming impact they will have over the 100 years following their emission. Emissions factors, calculated from each gas’s radiative force and lifespan in the atmosphere, predict the warming that an emission of a given gas will bring about over that time horizon. The GWP100 emissions factor for methane is 21 (IPCC, 2014), meaning that over 100 years, a unit-weight of emitted methane exerts 21 times the amount of warming as the same unit-weight of carbon dioxide. With the assistance of these factors, emissions of different GHGs can be compared to those of CO_2_ and communicated in the *carbon equivalence* unit, CO_2_e: one tonne of methane would be represented as 21 tonnes of CO_2_e. GWP100 has become the de facto emissions metric in national and international climate accounting and governance (Lynch, Cain et al., 2020). It is the foundation for reporting on Paris Agreement commitments and is used in emissions trading arrangements like the EU’s Emission Trading System.

**Figure 1.**
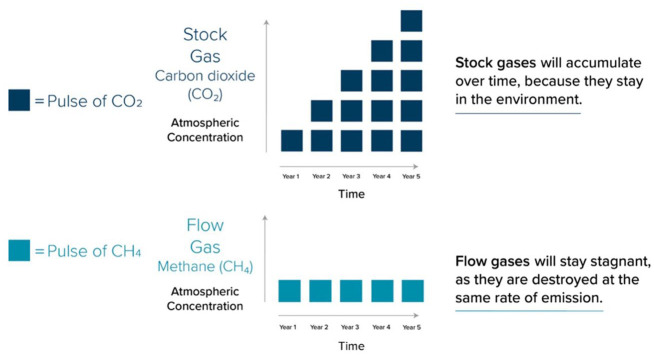
Stock and flow gases (from Mitloehner et al., 2020).

Advocates for the GWP* are concerned that the standard GWP100 metric fails to account for how the emissions *rates* of different GHGs interact with the climate (Allen et al., 2018; Lynch, Cain et al., 2020). The science behind these criticisms is not controversial and predates the GWP* metric by some margin (e.g. Lauder et al., 2013; Pierrehumbert, 2014). Due to their atmospheric half-life, some short-lived gases like methane exert their climate forcing impact over a relatively short period of time. For methane, this is around 10 years. If the rate of emissions stays *stable*, then it causes negligible *additional warming* (even if those emissions are very high) because the gases are removed at the same rate as they are replenished. Other gases, however, exert their radiative force over much longer periods. Carbon dioxide, for example, stays in the atmosphere and exerts its warming influence for thousands of years, and nitrous oxide for around 120 years. For timescales relevant to warming mitigation strategies, the emissions of these longer-lived gases continually add to the atmosphere stock. Their climate forcing impact is cumulative. Figure 1 emphasizes the ‘flow’ of short-lived climate pollutants relative to the accumulation of long-lived climate pollutants:

The authors of the original paper on GWP* compare the methane emissions of cows to ‘closed power stations’ to help make the distinction between the warming behaviours of long-lived and short-lived climate pollutants clear:

A power station emits CO_2_ by burning fossil fuels. … When it shuts down, it emits no more CO_2_ …. However, the CO_2_ already emitted continues to affect the climate for potentially thousands of years. So even after closing down, that power station still contributes to holding up global temperatures. Not so the cows. … If the herd remains the same size, with the same methane emissions every year, it will maintain the same amount of methane in the atmosphere year on year. In terms of its contribution to warming, this is equivalent to the closed power station. The power station pushed up global temperatures when it was running in the past, just as the farmer’s great-grandparent pushed up global temperatures when they were building up the herd of cattle. But neither a steady herd of cattle nor a defunct power station is pushing up global temperatures anymore. (Cain, 2018)

GWP100 cannot account for this dynamic because it does not take emission rates or historical emissions into account. GWP* navigates this issue by comparing *pulses* (in tonnes) of long-lived climate pollutants with *rates of emission* (in tonnes-per-year) of short-lived climate pollutants. With this ‘pulse-step comparison’, GWP* can identify instances in which herds of cattle, like closed power stations, have negligible warming impact: when the herd remains the same size, or where their methane emissions are otherwise maintained at a constant rate (see Folkers & Opitz, 2022 for discussion on methane-reduction bovine bioengineering projects). The metric also articulates how a reduced rate of emissions will confer *cooling*, as emissions are *removed* from the atmosphere faster than they are replenished (Cain et al., 2019). The difference in methodologies between the metrics is manifest in their respective names. Whereas the GWP100 metric uses a specific time horizon (100 years) to compare the warming impacts of all the GHGs, GWP* changes its calculations depending on which GHG is being evaluated, and so uses an asterisk or star to denote its variable calculations. This is what ‘when you wish upon a star’ in this paper’s title refers to: All the activity and disagreement that has come about from the way different metrics perform their calculations can be distilled into a single character.

Since its initial presentation (Allen et al., 2016, 2018), the GWP* metric has been the subject of several academic open-letter pieces and research papers. These have looked to refine the way GWP* calculates the warming impacts of the different gases (Cain et al., 2019; Smith et al., 2021), to outline what GWP* means for ‘zero emissions’ policies (Cain & Lynch, 2020; Lynch, Cain et al., 2020), and to examine its application in the reporting of the warming impacts of aviation (Lee et al., 2021) and livestock (Lynch, Järvenranta et al., 2020; Ridoutt, 2021). Critics suggest that in focussing on rates of emissions, the metric has a potential grandfathering problem. Under GWP*, pre-existing high emitters of short-lived climate pollutants can justify continuing these emissions by claiming that doing so exerts no additional warming. Those who have historically produced lower levels of short-lived climate pollutants, however, will either be locked into their low emission rates or be responsible for dramatically high warming impacts if their emissions increase (Rogelj & Schleussner, 2019). The other criticism relates to its use in governance mechanisms designed around GWP100. If emission trading schemes (like the European Trading Scheme) and emission reduction targets (like the Nationally Determined Contributions in the Paris Agreement) have been developed around one metric, it is unclear what impact the adoption of a new metric would have, and whether those impacts would be fairly distributed across national and sub-national actors (Schleussner et al., 2019). In response, others have claimed that GWP* has good fit with the Paris Agreement, not least because of the Agreement’s focus on warming, rather than emissions *per se* (Collins et al., 2020).

The scientists behind GWP* have used the agricultural sector – and livestock farming in particular – to demonstrate how GWP100 can affect the distribution of warming culpability. Modelling research evaluating the impact of enteric methane from ruminant animals like cattle and sheep has precipitated sustained scrutiny on the sustainability of meat and dairy consumption (e.g. Poore & Nemecek, 2018; Willett et al., 2019). These analyses use GWP100 to compare the warming impact of different emissions. If such studies have mainstreamed concern around the sustainability of meat and dairy (Gheihman, 2021), so this line of reasoning goes, then these concerns are predicated on an exaggerated evaluation of the warming impacts of livestock agriculture.

The GWP* authors are careful to point out that they are not arguing against concern about short-lived climate pollutants. But they stress that it is the warming impacts of long-lived climate pollutants – particularly carbon dioxide from the combustion of fossil fuels – that need to be targeted most urgently (Lynch et al., 2021). This is because stable emission rates of short-lived climate pollutants like methane are compatible with *warming*-neutral targets, whilst the continual emission and accrual of stock gases like CO_2_ and NO_2_ in the atmosphere are not. The authors of the metric have made this argument as keynote speakers at agricultural conferences (Oxford Farming Conference, 2021) and in industry publications (Irish Farmers Journal, 2020). This participation has had the (potentially intended) effect of enlisting the agricultural sector into a campaign to raise the profile of GWP* and to generate public support for its use in consequential climate governance.

For some in the livestock sector, GWP* has been seen as the vital exculpatory evidence to be used in the ongoing public trial of their sustainability credentials. GWP100 has created a ‘bogus burger blame’ (Mitloehner, 2021), they argue, a ‘methane myth’ that when debunked would show how ‘cows aren’t responsible for climate change’ (Stocks, 2019). For its advocates, GWP* shifts the focus back onto the emitters of longer-lived climate pollutants like the energy and transport sectors – with suggestions in the press that ‘new methane math takes the heat off the cows’ (Darigold, 2021) with ‘profound implications’ for the sector (Beef Central, 2020).

As an alternative to GWP100, GWP* quickly became a matter of concern rather than one of fact (Latour, 2004). The metric is not, in other words, just an analytic tool produced by the scientific community to provide some representation of the world (a matter of fact), it has become a social artefact, interacting with politics and societal values. As with the issues of livestock sustainability more generally (Kristiansen et al., 2021; Maye et al., 2021; Sanford et al., 2021), its cultural sensitivity is manifest in the strident tone of discussions in public fora like Twitter, Facebook and industry publications. Here, the GWP*/GWP100 issue is not presented as an evaluation of the relative technical merits of the two metrics, or of the mathematics best equipped to track the warming caused by different types of emission. Instead, it is about the metrics’ sources, the ethical imperatives to eat or boycott particular foodstuffs, and the distribution of warming culpability. GWP* has bled into more longstanding disagreements about the permissibility of meat in sustainable diets, the naturalness of livestock in agricultural systems and a nostalgia for traditionally farmed landscapes (Cusworth et al., 2022). As a scientific controversy, and as a function of the political and social sensitivity of the metric, the disagreement surrounding GWP* is not of the type that can be ‘resolved’ by further scientific inquiry (Kitcher, 2000).

## Reflexive performativity

What does the controversy tell us about the politics of environmental and metrical governance? The answer lies in a process we term *reflexive performativity.*

The warming footprint of agriculture – particularly livestock agriculture – is subject to intense scrutiny. This pressure is exerted from several different sources. Consumer expectations are shaping the purchasing policies of supermarkets and other large buyers, who are demanding emissions mitigation from the farming sector through sustainability purchasing policies and supply chain sustainability initiatives (Carolan, 2017; Freidberg, 2013). Although the agricultural sector has largely been excluded from emission trading schemes, an expanding market for offset credits, embodied in land-use changes like afforestation or soil carbon sequestration, is now providing incentive for farmers to strategically engage with mitigation activities. Policy developments are meshing with these commercial forces. Net-zero commitments made by the UK government, subnational actors like county councils, and industry bodies like the National Farmers’ Union (NFU) are creating new impetus for actors up and down the value chain to measure and manage their emissions. The UK’s new Environmental Land Management scheme, which is being designed to subsidize public goods such as emissions mitigation, is set to be the central policy mechanism through which these demands are realized in the UK. Like the coupling of financial and emissions accounting (Lovell & MacKenzie, 2011; Lovell et al., 2013), engagement with agri-environmental and emission mitigation activities is increasingly important to running a financially viable farm business (Cusworth & Dodsworth, 2021).

As the food system involves heterogenous landscapes and management practices, much of the (un)sustainability of agricultural practice is invisible (Carolan, 2006). Thus, strategies for governing the environmental footprint of agriculture’s complex supply chains rely heavily on metrics that can be deployed across geographies to standardize activities and measure performance (Dauvergne & Lister, 2012; Freidberg, 2013; Henson & Reardon, 2005). As with attempts to improve agriculture’s profitability (Miles, 2019), soil care (Johnston & Poulton, 2018) and the ability to ‘feed the world’ (Carolan, 2017), metrics are central to attempts to mitigate the sector’s emissions. They help make visible aspects of the food system that might otherwise be invisible, from warming impact to working conditions. Such metrics help stabilize dynamic information, allowing it to travel around the world whilst preserving its epistemological authority (Latour, 1983).

**Figure 2.**
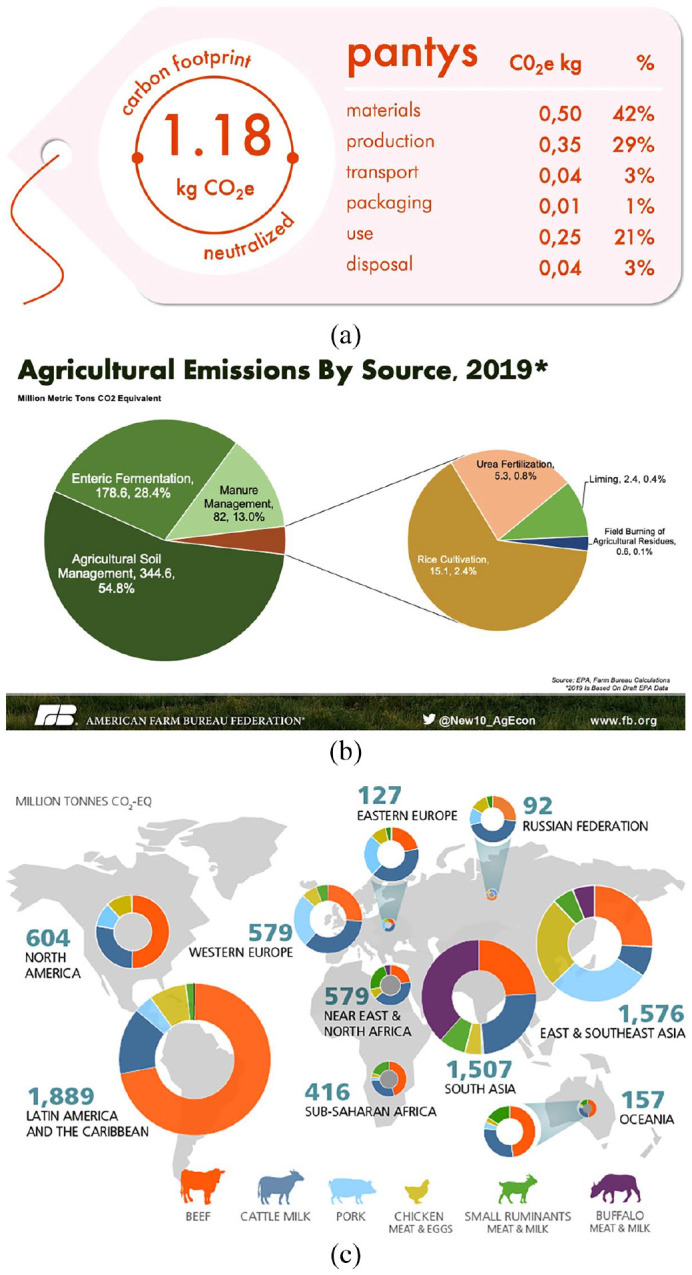
Three illustrations depicting agricultural emissions (in carbon equivalence) at different scales. (a): An example of a carbon footprint food label (Clever Carbon, 2022). (b): An American Farm Bureau Federation (2019) graphic breaking down national agricultural emissions. (c) An Food and Agriculture Organization (FAO, 2022) chart of regional emissions from different livestock sources.

Figure 2 shows a collage of visual representations of GHG impact, many of which rely explicitly or implicitly on metrics like GWP100. The ecolabel in Figure 2, for example, uses the metric’s aggregate CO_2_e unit to nudge consumers towards more sustainable consuming habits. The *This world in data* infographic, which is emblematic of the way information about the warming impacts of different foodstuffs is packaged and broadcast to wide audiences, communicates its analysis in the same way. As a result, it is not necessarily the emissions associated with a particular product or farm system *per se* that is important. It is the way that information is recorded and communicated by the GWP100 metric. Warming metrics are performative, in other words, as they shape the perceptions of politicians, industry bodies and consumers about the sustainability credentials of different foodstuffs or farm systems. This, in turn, influences the alacrity with which policy makers (through subsidies, taxes, etc.) and consumers (through public pressure to divest from livestock businesses, product boycotts, etc.) manage agricultural emissions. Ormond and Goodman (2015) reach the similar conclusions in their study of the communication of ‘everyday supply chain carbon’, finding that GWP100 and its CO_2_e unit have become extremely commercially and politically sensitive.

The demonstrations that the authors of GWP* have provided of the influence metric design has on the distribution of warming responsibility (e.g. Lynch et al., 2021; Lynch, Järvenranta et al., 2020) have alerted the sector to the availability of credible alternative methodologies for GHG commensuration. In so doing, the publication of GWP* and its subsequent interest in agricultural media publications has revealed both the strength and the contingency of the performativity of GWP100′s metric power. Strong, in the sense that it has clear structuring effects on the perceived sustainability of different foodstuffs. Contingent, in the sense that these calculations might be done otherwise, potentially producing radically different evaluations of the warming associated with a given foodstuff or farm system.

This power and contingency were made explicit by an interviewee speaking from an environmental NGO with a major voice in farming and land use policy debates. They explained that they had compared the emissions of a dairy farm they work with, using the GWP100 and GWP* metrics. Under the GWP* metric the farm was very nearly operating at a net-zero warming level. Under GWP100, the farm’s warming impact was substantial. One metric would demand concerted emissions-reductions activities to bring its emissions into check while the other would legitimize a business-as-usual model. Scaling this up to the sector at large, as has been a motif in the GWP* controversy (e.g. Darigold, 2021; Oxford Farming Conference, 2021; The Cattle Site, 2020), the adoption of one metric would demonstrate the sustainability of particular livestock systems while the preservation of the other would perpetuate its poor environmental reputation. The technical discussions of emissions and pulse-step comparisons overlie a more existential anxiety about the sector’s viability in a commercial and political atmosphere defined by emissions mitigation activities.

The livestock industry was already smarting from what it believes to be a sustained and inaccurate representation of its emissions profile. As McGregor et al. (2021) note, a 2006 report by the United Nations Food and Agriculture Organization (FAO), *Livestock’s Long Shadow* (FAO, 2006) features heavily in the story the sector tells of this misrepresentation. The report equated emissions from the livestock and transport sectors – inconsistently using a full life-cycle analysis to calculate livestock emissions and only tailpipe emissions for the transport sector. Over the course of the interviews, the report was repeatedly raised, particularly by those from within the industry. It served as a reminder that the issues of metrics and calculative specifics like GWP*/GWP100 can have consequences for public perception that are hard to shake, and that preventative steps need to be taken at the earliest possible opportunity to ward off similar problems in the future.

Attempts to influence the metrological emissions regime have taken several different forms. Industry bodies including Beef and Lamb New Zealand, the National Sheep Association, the Meat Industry Association, and farming unions from England, Scotland, Wales, and Northern Ireland have submitted a joint letter calling on the IPCC to use GWP* in the production of its evaluations of the warming impacts of different emitters (National Farmers Union [NFU], 2020). A European Commission call for evidence on a new ‘Methane Strategy’ gathered responses from multiple actors in the agricultural sector concerning how the GWP100 metric calculates the warming impacts of methane, and the merits of using GWP* in its stead (see European Commission, 2020). These include submissions from Dairy Industry Ireland, filed by the lobby group IBEC, the Irish Farmers Association, the European Dairy Association, and the CLEAR research institute at the University of California, Davis; the last of these elsewhere published a report for the Californian dairy industry on the relevance of GWP* for meeting climate neutrality targets (Mitloehner et al., 2020).

Beyond this direct lobbying, early interest in the metric following its presentation for the COP24 in 2018 (e.g. Cain, 2018) has spawned academic support from those beyond its original authors. This includes those with affiliations to the beef and dairy industries (e.g. Capper, 2020) writing about how the metric might challenge mainstream criticism of animal agriculture’s sustainability. These themes have been picked up by industry publications (Beef Central, 2020; Darigold, 2021; Farmers Weekly [FWI], 2019) and at industry events (Oxford Farming Conference, 2021), with messages about how the sector has been unfairly demonized by the GWP100 metric and that GWP* can help construct a new and more favourable narrative. Surveying the strategic efforts being deployed to raise the metric’s cultural and scientific profile, one interviewee working as a consultant in sustainable agricultural systems, explained the diverse set of actors who might have reason to put their shoulder to the wheel:

The push for policy to change in terms of GWP* will come partly from farmers who are outraged that they’re being measured using the wrong metric, but also from retailers. Even the best retailers like Waitrose and M&S [upmarket UK-based supermarket chains] aren’t requiring carbon footprinting yet. They’re looking at it … [and] if Waitrose or M&S started looking at their beef supply chain or the dairy supply chain, monitoring it and carbon footprinting it, using GWP* to communicate it with their customers could be a very positive narrative, that they could then pitch into influencing government policy over time. Groups like McDonald’s and so on, are looking at this pretty hard as well at a global level.

Lidskog (2014) notes that the involvement of parties with significant institutional and scientific credibility can amplify controversies; indeed, livestock-sector advocates for the GWP* metric do not miss an opportunity to mention their chosen metric’s origins at the University of Oxford (e.g. Agriland, 2020; Beef Central, 2020).

More generally, the performativity of these metrics is becoming subject to reflexive engagement and interaction. Decisions about metric design will shape the world these actors inhabit, and so, for them, there is strong incentive to shape the metrics at the soonest possible opportunity. This is the phenomenon of reflexive performativity. Having recognized the performativity of metrics in shaping the worlds they were designed to observe, these actors have learnt to be anticipatory rather than reactive in their dealings with the metrological regime they inhabit. This insight goes beyond the now-familiar conclusion that the ‘technologies of budgets, audits, standards, and benchmarks, apparently so mundane’ have become critical to ‘programs of governing at a distance … taking shape under rationalities of advanced liberalism’ (Rose et al., 2006, p. 95, glossing Power, 1995, 1997, 2000). It is about those who will be subject to these audits, standards, and benchmarks actively seeking participation in their design.

Like studies of biodiversity database management (Bowker, 2000a, 2000b) and organizational auditing (Pollock et al., 2018; Sauder & Fine, 2008), our account foregrounds the actor-oriented, rather than top-down or structuralist way that performativity exerts its influence (see Agrawal, 2005; Rose et al., 2006). Individual and nonhegemonic actors are seeking strategic and participatory intervention in the metrological regime they inhabit. In other regards, the phenomenon complicates pre-existing accounts of metrological performativity. When Bowker (2000a, 2000b) describes the bootstrapping problem (that cataloguing cannot proceed without pre-existing categories, and that initial candidates for category types overdetermine future cataloguing efforts) and the convergence problem (the ways diverse ways of recording, mapping and experiencing the world begin to homogenize), performativity is largely an unintended upshot of the makeshift nature of database design. Here, though, there is an intentionality at work.

Anderson’s (2010) influential account of anticipatory action can help us understand the nature of this intentionality. In a future characterized by risk and uncertainty (for some in the sector, the greater risk appears not to be the agronomic impacts of a changing climate but the political and consumer interventions being deployed in the name of its mitigation), those involved in the GWP* controversy are attempting to legitimize contemporary actions by relating them to possible future scenarios. If GWP* is adopted, the sector’s good environmental standing will be shored up in the future. If GWP100 remains the de facto warming metric, the sector can expect sustained scrutiny of its warming footprint. In framing the issue of metric selection in this way, and advocating for the uptake of GWP*, the activities of those involved in the controversy fall squarely into Anderson’s *pre-emptive* logic (see also Cooper, 2006). As Bowker (2000a) has written, many trajectories of convergence are understood to be ‘irreversible’: once they start, they cannot be unravelled. For many in the animal agriculture sector, GWP* provides an urgent, and creative, solution to the problem of pre-empting the irreversible effects of GWP100.

The emergence of the reflexive performativity is time sensitive. In the context of climate governance, the performative powers of metrics have been visible for long enough for actors have become aware of the diverse ways that they exercise power. Furthermore, it is only now that the availability of alternative methodologies and alternative calculations of the distribution of climate culpability have been publicized enough that there is good reason for certain actors to want to shape the metrological regime. To return to the framing used above, the creation and publicity of the GWP* metric has made visible both the *strength* and *contingency* of the metric power wielded by warming metrics. Precisely how long and precisely what sort of events are needed to provide ‘sufficient’ demonstration of the strength and contingency of metric power will vary between different cases. These are likely to be empirical issues rather than ones of universal abstraction and are worthy of further comparative study.

Marti and Gond (2018), writing in relation to financial performance measurements, describe the emergence of performativity as a sequential process. Individuals in an institution learn of some new auditing framework; they deploy it in experimental contexts; and gradually the institution and its actors change behaviours to better fit the framework’s predictive insights. Although the steps involved are different, the same *awareness-raising* dynamic can be seen in the case of reflexive performativity. First, actors must be aware of the metrics’ performative power (in this case, the mediating role metrics play in the political and commercial interventions designed to measure and minimize emissions). Second, they must be made aware of the contingency of metric design, most likely through the development of some alternative methodology (in this case, the publication of GWP*). This is because most actors lack the tools required to develop their own alternative metrological regime and the perceived intellectual authority needed to make it credible. And third, they must be aware that the adoption or preservation of one or other metric stands to confer or maintain some commercial or political advantage (in this case, the way GWP* can be used to downgrade the consumer and political concern directed towards the high warming footprint of meat and dairy). With these conditions in place, there is good strategic reason to reflexively engage with the metrological regime and whatever decision-making process shapes it.

This time-sensitive, awareness-raising process is manifest in the following excerpt from an interview with a member of a sustainable livestock advocacy group which has been vocal in advocating for the GWP* metric:

But when they came in with their GWP*, it demonstrated that because of the half-life of methane, it wasn’t a problem in a stable system. I think that was a very important breakthrough from my perspective. It just demonstrates that by looking at nature and the carbon cycle and the methane in that context there, what we’re talking about is not a problem.

## Reflexivity and reflexive modernization

What exactly are we referring to when we use the term ‘reflexivity’? Beck developed the idea to understand the emergence of a society preoccupied with managing the risks created by the technologies and activities of advanced modernity (Beck, 1992). For him, the apotheosis of modernity comes in the form of industrializing societies seeking to maximize the production of goods through the strategic development of modern technologies and the attendant rationales of measurement, enumeration, auditing, and incremental efficiency gains. Reflexive modernity arises because of the risks and uncertainties that modern society has precipitated: the ‘critical mass of side-effects’ from the ‘boundary-shattering force of market expansion, legal universalism and technical revolution’ (Beck et al., 2003, p. 2). These ‘externalities’ include the environmental and climate crises, and a suspicion over the claims to objectivity that have, since the Enlightenment period, accompanied the production of scientific knowledge. As the markers that were established as the telos of modern society remain elusive, the tools that were put forward to help attain its goals have become subject to suspicion. The process of modernity, Beck writes, ‘has begun to modernize its own foundations … it has become directed at itself’ (Beck et al., 2003 p. 1).

Latour’s (2003) critique of reflexive modernity and Beck et al.’s (2003) response help refine their cautiously shared version of what ‘reflexivity’ might mean in the context of reflexive modernization. For both Latour and Beck, it refers to the ways in which the technical and intellectual principles that were central to the first phase of modernization have become subject to the sort of scrutiny and rigour they were once used to animate: a reflexive engagement with, for example, the claims to objectivity associated with empiricism and the scientific method. They argue that because of the complex causal webs ubiquitous across modern life (the *networks* in Actor-Network Theory), the concept of reflexivity relates to how the ‘unintended consequences of actions reverberate throughout the whole of society in such a way that they have become intractable’ (Latour, 2003, p. 36). Being reflexive, then, is not about a mastery of some socio-technical *thing.* Nor is it a move towards a more complete understanding of the social and political milieu from which the thing came. It is about a recognition that such objectivity is impossible, and that acting in modern society, in and amongst contemporary crises like climate change and pandemics, has implications that ripple out in uncertain ways that can never be fully grasped (Latour, 2003).

The same reflexivity colours the engagement with the warming metrics we describe above. True to Latour’s definition above, the industry actors who have reflexively engaged with the GWP* metric have not championed the metric solely on the premise that it takes climate governance closer to some objective metrological and epistemological framework. The claim, instead, is that a metric’s performative powers need to be an active consideration in the design of the interventions used to manage the pervasive risks of modern life (like climate change). Metrics, to take this back to Cooper’s critical metrology, are political. The angst that exists about their usage and design – manifest in the GWP* controversy – arises as a combined function of two observations: first, that metrics are a proxy for life that can never be, and should not be assumed to be, objective, and second, that their adoption has impacts that reverberate out across complex webs linking, in the case of food, farmers, consumers, government agencies, international climate governance organizations, supermarkets and so on. That warming metrics are political issues of concern can be seen in the institutional response to the new metric. In the report filed by Working Group 1 (who deal with the ‘physical science’ basis for climate change) for the IPCC’s 2021 6^th^ Assessment Report, the touchy subject of warming metric design and selection is discussed:

New emission metric approaches like GWP* … relate changes in the emission rate of short-lived GHGs to equivalent cumulative emissions of CO_2_ …. It is a matter for policymakers to decide which emission metric is most applicable to their needs. This Report does not recommend the use of any specific emission metric as the most appropriate metric depends on the policy goal and context. (IPCC, 2021, pp. 66-67)

The mainstay of Latour’s (2003) evaluation of Beck’s reflexive modernization is that it needs to be exposed to empirical scrutiny. It is a good idea, he writes, but how can we know if it is true? He provides several shovel-ready tests that might be deployed to verify its accuracy as a grand and totalizing sociological theory. One of the tests concerns the extent to which objects (matters of fact) have become quasi-objects (matters of concern). If a reflexive modernism were underway, warming metrics like GWP* and GWP100 would no longer be ‘incontrovertible, bounded, mastered, black-boxed, expert-ruled innovation[s]’, but quasi-objects that get presented ‘with their unwanted consequences and with their uncertain and puzzled makers and users attached’ (Latour, 2003 p. 43).

The ‘makers’ (the scientists who developed the metric and who have appeared in farm industry events and publications) and would-be ‘users’ (e.g. the signatories to the joint letter to the IPCC advocating for the uptake of GWP*) have sought to attach themselves to the metric. The controversy the metric has precipitated can be read as an exercise in this sort of attachment-making. That the ‘unwanted consequences’ have become attached to the metric can be seen in the way the IPCC Working Group 1 have approached the issue of alternative warming metrics like GWP*. There, a metric’s politics are indivisible from its technical and scientific substance. Deciding which metric to use or which time-horizon best tracks onto ‘dangerous anthropogenic warming’ is not reducible to an evaluation of the different metrics’ technical aptitude. It is a subject that needs to be exposed to some deliberative democracy in which the social, political, and environmental implications of the different metrics are interwoven with an analysis of its technical specifics. Although it is a far cry from the rigorous empirical verification for which Latour called, there is preliminary evidence that the object to quasi-object transition he suggests is underway in this case.

The STS focus on climate change governance has shown the proclivity for the issue to be presented in ‘intensely scientific’ terms (Wynne, 2010). In this corner of climate governance, though, this presentation has been unsettled by a recognition of the social and political implications of the tools used to make the changing climate a governable entity. We extend the call issued by Mahony and Hulme (2018) and taken up by Lahn (2021) to direct further study to track the evolving way the IPCC and its constituent Working Groups approach issues like metric design as objects or quasi-objects. For the time being, though, we present the phenomenon of reflexive performativity (of which the GWP* controversy is just one example) as the metrological expression of Beck et al.’s (1994) reflexive modernization.

## Conclusions

By paying close attention to the specifics of GWP* and GWP100, as per Cooper’s critical metrological framework, we have made explicit the sensitivity of metric design in environmental regulation to political, commercial, and cultural interactions. The study has revealed the phenomenon we term reflexive performativity. To return to Kula’s story of peasants, lords, and tithes, and to the question posed in the paper’s introduction, what has changed in the past 500 years? In a sense, very little. Metrics (be they containers for grain or global warming metrics) directly influence the activities of those who fall under their metrological jurisdiction. Certain actors – the lords of Iskrzynia and Kroscienko or the CEO of a large dairy company – might try and use this as a pressure point to leverage commercial or political advantage. For those whose lives stand to be affected, though, the metrics are also a plausible site for resistance, disgruntlement, and organization.

Writing about the impacts that GWP100 has had on the markets developed to manage anthropogenic warming, Cooper (2015, p. 1799) concludes that ‘once markets are active, dominant agents in the market are likely to resist shifting from the commensuration that was developed in their interest’. This conclusion is similar to those reached by Bourdieu in his description of how powerful actors leverage their economic, social, and cultural capital to preserve the social order in which they dominate (Bourdieu, 1988); and to those of social theorists using ideas of governmentality to describe how powerful actors use mundane tools of measurement to shore-up their power and exert their influence (Power, 1997; Rose et al., 2006). The conclusions of this paper, though, add some complexity to the situation. It is not just that dominant agents act to shape the metrical regime; but that actors of all scales and size have (rightly) identified metrics as central sources of power exercised in the governance of their activities. They have learnt, as a result, to be reflexive and anticipatory in their engagement with metrological performativity. The situation regarding GWP* is, at the time of writing, still live. But its resolution will not be shaped by just the dominant actors in this particular metrological regime, but all manner of groups who see GWP* as a way to have their say.
